# Associations of *BDNF* Genotype and Promoter Methylation with Acute and Long-Term Stroke Outcomes in an East Asian Cohort

**DOI:** 10.1371/journal.pone.0051280

**Published:** 2012-12-11

**Authors:** Jae-Min Kim, Robert Stewart, Man-Seok Park, Hee-Ju Kang, Sung-Wan Kim, Il-Seon Shin, Hye-Ran Kim, Myung-Geun Shin, Ki-Hyun Cho, Jin-Sang Yoon

**Affiliations:** 1 Department of Psychiatry, Chonnam National University Medical School, Gwangju, Korea; 2 Institute of Psychiatry, King's College London, London, United Kingdom; 3 Department of Neurology, Chonnam National University Medical School, Gwangju, Korea; 4 Brain Korea 21 Project and Center for Biomedical Human Resources, Chonnam National University Medical School, Gwangju, Korea; 5 Department of Laboratory Medicine, Chonnam National University Medical School, Gwangju, Korea; Universität Stuttgart, Germany

## Abstract

**Background:**

Brain derived neurotrophic factor (BDNF) has been shown to play an important role in poststroke recovery. BDNF secretion is influenced by genetic and epigenetic profiles. This study aimed to investigate whether *BDNF* val66met polymorphism and promoter methylation status were associated with outcomes at two weeks and one year after stroke.

**Methods and Findings:**

A total of 286 patients were evaluated at the time of admission and two weeks after stroke, and 222 (78%) were followed one year later in order to evaluate consequences of stroke at both acute and chronic stages. Stroke outcomes were dichotomised into good and poor by the modified Rankin Scale. Stroke severity (National Institutes of Health Stroke Scale), physical disability (Barthel Index), and cognitive function (Mini-Mental State Examination) were measured. Associations of *BDNF* genotype and methylation status on stroke outcomes and assessment scale scores were investigated using logistic regression, repeated measures ANOVA and partial correlation tests. *BDNF* val66met polymorphism was independently associated with poor outcome at 2 weeks and at 1 year, and with worsening physical disability and cognitive function over that period. Higher *BDNF* promoter methylation status was independently associated with worse outcomes at 1 year, and with the worsening of physical disability and cognitive function. No significant genotype-methylation interactions were found.

**Conclusions:**

A role for BDNF in poststroke recovery was supported, and clinical utility of *BDNF* genetic and epigenetic profile as prognostic biomarkers and a target for drug development was suggested.

## Introduction

Stroke is frequently associated with significant disability and impaired quality of life. Adverse outcomes after stroke are believed to be determined by a series of biochemical, hemodynamic, and neurophysiologic changes. Neurotrophic factors play a central role in this process through their neuroplastic effects [1]. Brain-derived neurotrophic factor (BDNF), the most abundant neurotrophin within the brain, is important for poststroke recovery, since it promotes neuro- and angiogenesis in animals [2], [3]. A single nucleotide polymorphism (SNP) producing a valine-to-methionine substitution at codon 66 (val66met) in the *BDNF* gene is associated with reduced activity-dependent secretion of BDNF [4], and *BDNF* met/met mice have been found to exhibit greater deficits in poststroke locomotor functions and reduced angiogenesis [5]. However, associations in humans between the *met* allele and stroke outcomes have been less conclusive [6]–[9]. BNDF expression is also regulated by epigenetic chromatin remodeling, including DNA methylation of cytosines in cytosine-guanine (CpG) dinucleotides. An increase in CpG methylation at promoter regions on the *BDNF* gene have been found to be correlated with decreased neuronal synthesis of BDNF [10] and with an increased risk of bipolar disorder [11]. Based on these findings, it could be postulated that *BDNF* promoter methylation status will be associated with stroke outcomes, although this hypothesis has not been tested yet. Using data from a longitudinal study of a post-stroke cohort, we examined the roles of *BDNF* val66met polymorphism and promoter methylation status on outcomes at two weeks and one year after the index stroke.

## Methods

### Study overview and participants

This analysis was carried out as a component of a larger parent study, which seeks to investigate neurologic and psychiatric morbidity in stroke survivors using a naturalistic prospective design which has been previously described in detail [12]. Participants were consecutively recruited from all patients with recent ischemic stroke hospitalized within the Department of Neurology of Chonnam National University Hospital, Gwangju, South Korea. Stroke severity was assessed in all hospitalized patients at the time of admission (prior to treatment, usually within 6 hours of stroke onset) and treatment was carried out by the research neurologists based on the guidelines for the management of stroke [13] over the study period. About 2 weeks poststroke, all patients were approached regarding study participation. Inclusion criteria were: i) confirmed ischemic stroke by brain magnetic resonance imaging (MRI), or computed tomography (CT) if MRI was contraindicated; ii) ability to complete the necessary investigations and questionnaires; and iii) capacity to understand the objective of the study and provide informed consent. Exclusion criteria were: i) severe physical illnesses which were life-threatening or interfering with the recovery from stroke; ii) communication difficulties due to dysphasia or dysarthria precluding informed consent and questionnaire completion; iii) any of the following comorbid neuropsychiatric conditions: dementia, Parkinson's disease, brain tumor, epilepsy, psychoses, alcohol and substance dependence; iv) severe physical illnesses limiting movement prior to stroke; and v) Mini-Mental State Examination (MMSE) [14] score of <16. The recruitment period was from 2006 to 2010 and attempts were made to follow up all participants after one year. Overall, assessments were made at the time of admission, 2 weeks and 1 year after the stroke to investigate, in the context of the wider study, a broad range of consequences of stroke at both acute and chronic stages. All participants gave written informed consent and the study was approved by the Chonnam National University Hospital Institutional Review Board.

Of 423 patients eligible and consenting to participate in the study, 286 (68%) agreed to provide blood samples and formed the sample for this analysis. There were no significant differences between participants and non-participants with respect to any demographic and clinical characteristics (all p-values>0.1). At 1 year, 222 (78%) were followed up (MMSE was available in 201). Those present or not at 1 year did not significantly differ at baseline with respect to any demographic and clinical characteristic (all p-values>0.1). The mean (SD) time from at admission to at 2 week assessment points were 12.3 (3.0) days and to at 1 year were 13.2 (3.6) months.

### Evaluations for stroke severity

Global disability was evaluated by the modified Rankin Scale (mRS) [15], the scores of which range from 0–6 with higher scores indicating more severe disability. Stroke severity was measured using the National Institutes of Health Stroke Scale (NIHSS; scores ranging 0–42 with higher scores indicating more severe pathology) [16], physical disability was measured with the Barthel Index (BI; scores ranging 0–100 with lower scores indicating more severe disability) [17], and cognitive function was evaluated by the MMSE (scores ranging 0–30 with lower scores indicating lower cognitive function). Scores on the mRS, NIHSS, and BI were obtained at admission, at 2 week and at 1 year assessment points, while the MMSE scores were available at the 2 week and at 1 year evaluation points.

### BDNF genotyping and DNA methylation analysis

**Figure 1:**
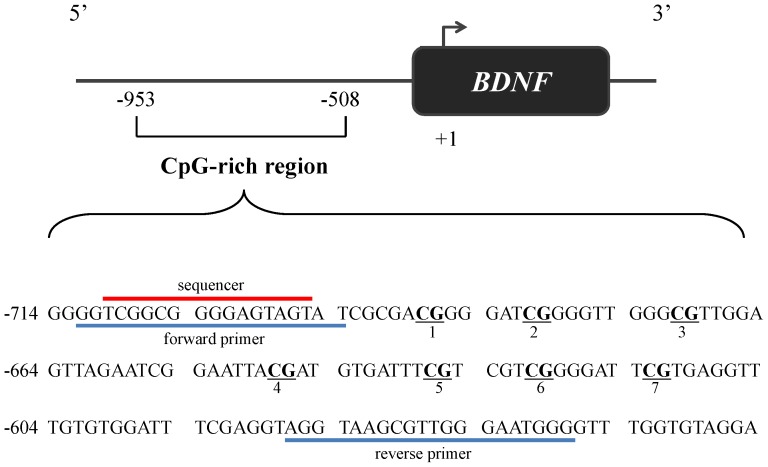
*BDNF* promoter regions for analyzing methylation status. Of the CpG-rich region of *BDNF* promoter, the portion analyzed by bisulfite pyrosequencing is shown. The CpGs are in underlined and bold font and numbered. Forward and backward primers and sequencer are designated. Numbering of the gene sequence is relative to the transcriptional start site.

Blood samples were obtained in a subsample who agreed to this. DNA was extracted from venous blood, and genotyping and DNA methylation analysis were conducted using standard procedures. For genotyping, polymerase chain reaction (PCR) and the PCR-based restriction fragment length polymorphism (RFLP) assays were performed. The primer sequences used were the forward primer 5′-ACTCTGGAGAGCGTGAATGG-3′ and the reverse primer 5′-ACTACTGAGCATCACCCTGGA-3′. The amplification conditions were pre-denaturation at 95°C for 5 minutes, followed by 40 cycles consisting of denaturation at 95°C for 30 seconds, 62°C for 30 seconds and 72°C for 30 seconds, and post-elongation at 72°C for 5 minutes, with a final maintenance step at 4°C. The PCR products were digested at 37°C with the corresponding restriction enzyme (*Eco*72I), and gel electrophoresis was used to detect the 196G (*val*: 99 and 72 bp fragments) and 196A (*met*: 171 bp fragment) alleles. The *BDNF* promoter region for analyzing methylation status is presented in Figure 1. These data have been deposited in GenBank (accession number: BankIt1568919 BDNF JX848620). A CpG-rich region of the promoter between −694 and −577, relative to the transcriptional start, including seven CpG sites were analyzed as in other studies [18], [19]. Genomic DNA (1 µg) was extracted from leukocytes using QIAamp DNA Blood Mini Kit (Qiagen, Valencia, CA, USA) following the manufacturer's suggested protocol, and was bisulfite- treated using the EpiTech Bisulfite Kit (Qiagen, Valencia, CA, USA) according to the manufacturer's protocol. A 118 bp fragment of *BDNF* promoter was amplified by PCR from bisulfite-treated DNA using the forward and reverse primers designated in Figure 1. PCR conditions were 95°C for 15 minutes, followed by 45 cycles of 95°C for 15 seconds, 57°C for 30 seconds, and 72°C for 15 seconds, with a final extension of 5 minutes at 72°C. PCR products were sequenced using the PSQ 96M Pyrosequencing system (Biotage) according to the manufacturer's protocol with the following sequencing primers designated in Figure 1. The methylation percentage at each CpG region was quantified using the Pyro Q-CpG software, version 1.0.9 (Biotage). The genotype was categorized as ‘*val/val*’, ‘*val/met*’, and ‘*met/met*’. The individual methylation percentages at seven CpG sites of a promoter region and their average values were estimated.

### Demographic and clinical covariates

Those characteristics potentially associated with stroke outcomes were considered as covariates in this analysis. Age, gender, year of education, previous history of stroke, and comorbidity with heart disease, hypertension and diabetes were recorded according to information obtained from the participant or their caregiver, or by medical records, as appropriate. Stroke location by hemisphere was divided into left, right, and bilateral using the brain MRI or CT imaging. Blood pressure, and serum glucose and total cholesterol levels were measured. Depressive symptoms were evaluated by the Hamilton Depression Rating Scale (HAMD) [20], scores ranging 0–52 with higher scores indicating more severe pathology.

### Statistical analyses

Patients were dichotomized into two groups at each evaluation point by applying the mRS score cut-off 1 (no significant disability)/2 (slight disability). Demographic and clinical characteristics, scores on NIHSS and BI at admission, and HAMD scores at 2 weeks were compared according to the mRS score cut-off at admission using t-tests or χ^2^ tests as appropriate. To compare the *BDNF* genotype distribution and methylation percentages between patients with good outcome (mRS grade≤1) and poor outcome (mRS grade≥2) at 2 weeks and 1 year, pairwise and multiple logistic regression analyses were conducted adjusting for those demographic and clinical characteristics at admission which were associated with the mRS outcome (p-value<0.1). The interactive effects of *BDNF* genotype and methylation percentages on the outcomes were investigated using multivariable logistic regression models with the same adjustment. To investigate the associations with scores on stroke assessment scales (NIHSS, BI, and MMSE) across the evaluation points, repeated measures ANOVA tests for *BDNF* genotype and partial correlation tests for *BDNF* methylation percentages were conducted adjusting for those demographic and clinical characteristics at admission which were associated with the mRS grades (p-value<0.1). Additional analyses were carried out to compare those demographic and clinical characteristics at admission which were associated with the mRS grades (p-value<0.1) by *BDNF* genotype and tertiles of CpG average methylation percentage, and to investigate the associations between *BDNF* genotype and promoter methylation percentages. Statistical analyses were carried out using SPSS 13.0 software.

## Results

### Demographic and clinical characteristics

Overall distributions of sample characteristics are summarized in the first column of Table 1. These characteristics are compared by mRS grades at admission in the 2^nd^–4^th^ columns of Table 1. Compared to those with mRS≤1 (N = 98), the group with mRS≥2 (N = 188) were older and had higher scores on NIHSS, BI, and HAMD (all p-values<0.1). There were no significant differences in the mean values of these characteristics by *BDNF* genotype and tertiles of CpG average methylation percentage (Table 2).

**Table 1:** Baseline characteristics by modified Rankin Scale (mRS) grades at admission.

	All patients (N = 286)	mRS≤1 (N = 98)	mRS≥2 (N = 188)	p-value^a^
Age, mean (SD) years	64.5 (9.5)	63.1 (9.4)	65.2 (9.5)	0.087
Gender, N (%) male	169 (59.1)	63 (64.3)	106 (56.4)	0.197
Education, mean (SD) year	8.5 (5.0)	8.9 (4.9)	8.3 (5.1)	0.319
Previous stroke, N (%)	28 (9.8)	8 (8.2)	20 (10.6)	0.504
Heart disease, N (%)	27 (9.4)	13 (13.3)	14 (7.4)	0.110
Hypertension, N (%)	141 (49.3)	44 (44.9)	97 (51.6)	0.282
Diabetes, N (%)	84 (29.4)	30 (30.6)	54 (28.7)	0.739
Stroke hemisphere, N (%)				
Left	130 (45.5)	49 (50.0)	81 (43.1)	0.495
Right	141 (49.3)	45 (45.9)	96 (51.1)	
Bilateral	15 (5.2)	4 (4.1)	11 (5.9)	
Systolic blood pressure, mean (SD) mmHg	138.5 (21.0)	138.0 (25.6)	138.8 (18.2)	0.801
Diastolic blood pressure, mean (SD) mmHg	84.2 (15.1)	82.2 (16.2)	85.2 (14.5)	0.111
Glucose, mean (SD) mg/dL	148.4 (69.2)	150.3 (77.4)	147.4 (64.6)	0.736
Total cholesterol, mean (SD) mg/dL	188.7 (38.9)	191.2 (38.5)	187.4 (39.2)	0.444
National Institutes of Health Stroke Scale, mean (SD) score	3.4 (3.2)	1.1 (0.8)	4.6 (3.3)	<0.001
Barthel Index, mean (SD) score	80.1 (23.0)	97.6 (5.8)	71.0 (23.3)	<0.001
Hamilton Depression Rating Scale at 2 week, mean (SD) score	7.9 (6.3)	6.9 (6.1)	8.4 (6.3)	0.047

a p-value using t-tests or χ^2^ tests, as appropriate.

**Table 2:** Confounding factors at baseline by *BDNF* genotype and tertiles of CpG average methylation percentage.

	*BDNF* genotype				Tertiles of *BDNF* promoter CpG average methylation percentage			
	val/val (N = 66)	val/met (N = 157)	met/met (N = 63)	p-value^a^	Low (30–43%)	Middle (44–48%)	High (49–70%)	p-value^a^
Age	64.4 (10.1)	64.3 (9.4)	64.9 (9.2)	0.912	63.4 (8.9)	65.2 (9.9)	64.8 (9.7)	0.386
National Institutes of Health Stroke Scale	3.2 (3.4)	3.5 (3.0)	3.4 (3.3)	0.801	3.3 (2.5)	3.8 (3.6)	3.2 (3.2)	0.362
Barthel Index	81.0 (23.2)	79.2 (22.5)	81.4 (24.0)	0.777	79.5 (20.3)	78.7 (25.1)	82.1 (23.3)	0.565
Hamilton Depression Rating Scale	7.3 (5.9)	8.0 (6.5)	8.2 (6.0)	0.674	8.0 (6.4)	7.1 (5.9)	8.7 (6.6)	0.234

a p-value derived from ANOVA.

Data are mean (SD).

### BDNF genotype and promoter methylation by stroke outcome status

*BDNF* val66met polymorphism and promoter methylation percentages are compared by stroke outcome status (good outcome as mRS≤1, and poor outcome as mRS≥2) at 2 weeks and at 1 year in Table 3. No deviation from the Hardy–Weinberg equilibrium was observed for *BDNF* genotype (p-value = 0.097). Power estimates (calculated by an online program: http://www.dssresearch.com/KnowledgeCenter/toolkitcalculators/statisticalpowercalculators.aspx) of the allele test for *BDNF* val66met was 72%, and of the promoter methylation percentages were 93∼95%. Poor outcome at 2 weeks was significantly associated with higher frequency of the *BDNF met* allele and higher methylation percentage at *BDNF* promoter CpG site 6 after adjustment for age and scores on NIHSS, BI, and HAMD in the pairwise logistic regression analyses. Poor outcome at 1 year was significantly associated with higher frequency of the *met* allele, with higher methylation percentages at CpG sites 4 and 7, and with a higher average value in the pairwise logistic regression analyses with the same adjustment model. In the multiple regression analyses, poor outcome at 2 weeks was independently associated only with the higher frequency of the *BDNF* met allele; and poor outcome at 1 year was independently associated with the higher frequency of the *BDNF* met allele and with higher methylation percentage at CpG site 4 (Table 4). There were no interactive effects of *BDNF* genotype and methylation percentages on 2 week or 1 year stroke outcomes in the multivariate logistic regression models (Table 5). In addition, *BDNF* genotype was not significantly associated with *BDNF* methylation percentages (Table 6).

**Table 3:** *BDNF* genotype and promoter methylation percentages by stroke outcome status at 2 week and at 1 year.

*BDNF*	At 2 weeks post-stroke				At 1 year post-stroke			
	Good outcome (N = 163 )	Poor outcome (N = 123)	OR (95% CI)	p-value^a^	Good outcome (N = 146)	Poor outcome (N = 76)	OR (95% CI)	p-value^a^
Genotype, N (%)								
val/val	45 (27.6)	21 (17.1)	Ref		39 (26.7)	12 (15.8)	Ref	
val/met	86 (52.8)	71 (57.7)	1.97 (0.98–3.97)	0.018	80 (54.8)	41 (53.9)	1.65 (0.76–3.59)	0.038
met/met	32 (19.6)	31 (25.2)	2.75 (1.20–6.31)		27 (18.5)	23 (30.3)	3.14 (1.29–7.68)	
Methylation percentages, mean (SD)								
CpG 1	13.7 (3.5)	13.8 (3.5)	1.00 (0.93–1.08)	0.988	13.6 (3.5)	14.2 (3.9)	1.06 (0.98–1.15)	0.179
CpG 2	97.8 (7.0)	96.6 (10.0)	0.98 (0.95–1.01)	0.253	96.5 (9.7)	98.5 (5.7)	1.04 (0.99–1.09)	0.068
CpG 3	72.3 (6.3)	72.5 (6.4)	1.00 (0.96–1.05)	0.881	72.4 (6.1)	72.3 (7.0)	1.00 (0.96–1.05)	0.928
CpG 4	30.8 (7.5)	29.4 (6.8)	0.96 (0.93–1.01)	0.062	28.9 (6.5)	32.3 (7.5)	1.09 (1.04–1.15)	0.001
CpG 5	55.6 (12.8)	54.6 (10.1)	0.99 (0.97–1.01)	0.392	55.9 (12.5)	54.5 (10.9)	0.99 (0.96–1.01)	0.350
CpG 6	19.5 (16.4)	23.0 (18.2)	1.02 (1.00–1.04)	0.033	19.7 (15.7)	23.6 (19.7)	1.02 (0.99–1.03)	0.079
CpG 7	29.5 (21.9)	30.4 (19.2)	1.01 (0.99–1.02)	0.468	27.1 (20.2)	35.4 (21.3)	1.02 (1.01–1.04)	0.005
CpG average	45.6 (6.1)	45.7 (6.0)	1.01 (0.97–1.06)	0.660	44.9 (6.0)	47.3 (5.5)	1.08 (1.03–1.04)	0.003

a p-value using logistic regression likelihood ratio tests adjustment for age and baseline scores on National Institutes of Health Stroke Scale, Barthel Index, and Hamilton Depression Rating scale.

**Table 4:** Multiple regression analyses of *BDNF* genotype and promoter methylation on poor stroke outcomes at 2 weeks and at 1 year.

*BDNF*	At 2 weeks post-stroke		At 1 year post-stroke	
	OR (95% CI)	p-value^a^	OR (95% CI)	p-value^a^
Genotype				
val/val	Ref		Ref	
val/met	1.87 (0.92–3.78)	0.027	1.53 (0.69–3.37)	0.048
met/met	2.58 (1.12–5.95)		2.53 (1.01–6.38)	
Methylation site				
CpG 4	NA		1.09 (1.02–1.17)	0.011
CpG 6	1.01 (0.99–1.03)	0.051	NA	
CpG 7	NA		1.02 (0.99–1.05)	0.092
CpG average	NA		0.96 (0.86–1.07)	0.414

a p-value using logistic regression likelihood ratio tests adjustment for age and baseline scores on National Institutes of Health Stroke Scale, Barthel Index, and Hamilton Depression Rating scale.

**Table 5:** Multivariate analyses examining the interactive effects of *BDNF* val66met polymorphism and promoter methylation on poor stroke outcomes at 2 weeks and at 1 year.

	At 2 weeks (N = 286)		At 1 year (N = 222)	
	Wald	OR (95% CI)	Wald	OR (95% CI)
val66met polymorphism×CpG1	2.55	0.90 (0.79–1.02)	0.43	0.96 (0.84–1.09)
val66met polymorphism×CpG2	0.02	1.01 (0.94–1.08)	0.15	0.97 (0.85–1.11)
val66met polymorphism×CpG3	0.18	0.98 (0.91–1.07)	2.42	0.91 (0.81–1.03)
val66met polymorphism×CpG4	0.04	1.01 (0.94–1.08)	0.01	0.99 (0.92–1.08)
val66met polymorphism×CpG5	0.83	0.96 (0.89–1.05)	0.01	1.00 (0.96–1.05)
val66met polymorphism×CpG6	0.20	0.99 (0.97–1.09)	0.43	1.01 (0.98–1.04)
val66met polymorphism×CpG7	0.02	1.01 (0.98–1.02)	0.21	1.01 (0.98–1.03)
val66met polymorphism×CpG average	0.49	0.98 (0.91–1.05)	0.21	1.02 (0.94–1.10)

All data are adjusted for age and baseline scores on National Institutes of Health Stroke Scale, Barthel Index, and Hamilton Depression Rating scale.

**Table 6:** *BDNF* promoter methylation percentages by val66met polymorphism.

Methylation site	Mean (SD) methylation percentage by val66met polymorphism			p-value^a^
	val/val (N = 66)	val/met (N = 157)	met/met (N = 63)	
CpG 1	14.0 (3.6)	13.4 (3.5)	14.2 (3.2)	0.219
CpG 2	97.6 (7.9)	96.7 (9.9)	98.7 (2.0)	0.115
CpG 3	72..9 (6.7)	71.8 (6.9)	73.4 (4.1)	0.183
CpG 4	30.6 (7.9)	29.6 (7.8)	31.3 (4.8)	0.221
CpG 5	55.9 (11.3)	54.2 (11.3)	56.9 (13.1)	0.289
CpG 6	17.8 (14.9)	21.6 (17.9)	23.0 (17.6)	0.188
CpG 7	28.0 (18.4)	28.6 (19.9)	34.7 (24.6)	0.110
CpG average	45.3 (6.0)	45.4 (6.1)	46.9 (5.8)	0.116

a p-value derived from ANOVA.

### Adjusted associations with stroke assessment scale scores

**Figure 2:**
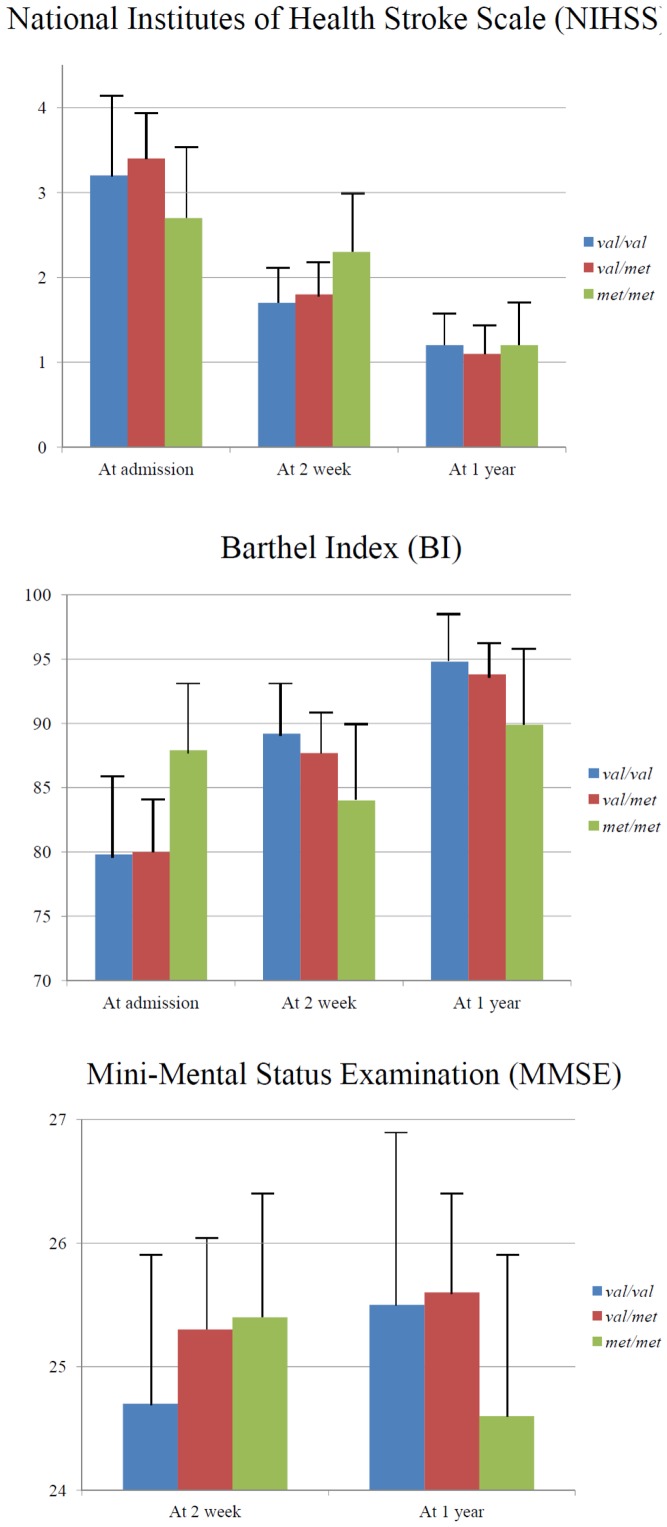
Comparing scores on stroke assessment scales between groups of *BDNF* val66met polymorphism over time. Repeated measures ANOVA demonstrating the following: For NIHSS, no group effect of genotype (p-value = 0.976) or group by time interaction (p-value = 0.259); For BI, no group effect of genotype (p-value = 0.985) but a significant group by time interaction (p-value = 0.003); For MMSE, no group effect of genotype (p-value = 0.826) but a significant group by time interaction (p-value = 0.035) after adjustment for age and Hamilton Depression Rating Scale score.

The mean (SD; range) scores on stroke assessment scales at three evaluation points are presented in the Table 7. Associations of *BDNF* val66met polymorphism with scores on the stroke assessment scales are illustrated in Figure 2. Repeated measures ANOVA demonstrated significant genotype group by time interactions on BI and MMSE scores after adjustment for age and HAMD score. That is, the ‘val/val’ and ‘val/met’ genotype groups showed improvement in their scores over 1 year after stroke, while the ‘met/met’ group displayed worsening or significantly less improvement. However, there were no significant main group effects of genotypes on the NIHSS, BI, and MMSE scores. Partial correlations of *BDNF* methylation percentages with baseline scores and changes at follow-up for stroke assessment scales adjustment for age and HAMD score are summarised in Table 8. With respect to BI, worsening scores from admission to 2 weeks were significantly associated with a higher CpG site 7 methylation percentage; and those from admission to 1 year were significantly associated with higher CpG site 7 and average methylation percentages. Worsening scores on MMSE from 2 weeks to 1 year were significantly associated with higher CpG site 2, 4 and 7, and average methylation percentages. No such associations were found with baseline scores on BI and MMSE, nor with any baseline/changed scores on NIHSS.

**Table 7:** Scores on stroke assessment scales at three evaluation points.

	At admission (N = 286)	At 2 weeks (N = 286)	At 1 year (N = 222)
National Institutes of Health Stroke Scale	3.4 (3.2; 0–18)	2.1 (2.2; 0–10)	1.1 (1.7; 0–7)
Barthel Index	80.1 (23.0; 0–100)	85.8 (18.4; 0–100)	93.1 (14.6; 25–100)
Mini-Mental State Examination		24.8 (4.0; 16–30)	25.3 (4.6; 3–30)^a^

a Data were available in 201 patients.

Data are mean (SD; range).

**Table 8:** Partial correlations between *BDNF* promoter methylation percentages and scores on stroke assessment scales.

Methylation site	Adjusted partial correlation coefficient between methylation percentages and outcome measures							
	National Institutes of Health Stroke Scale			Barthel Index			Mini-Mental State Examination	
	Score at admission (N = 286)	Change in score from admission to 2 weeks (N = 286)	Change in score from admission to 1 year (N = 222)	Score at admission (N = 286)	Change in score from admission to 2 weeks (N = 286)	Change in score from admission to 1 year (N = 222)	Score at 2 weeks (N = 286)	Change in score from 2 weeks to 1 year (N = 201)
CpG1	−0.006	−0.014	+0.031	−0.052	+0.020	−0.025	−0.075	−0.074
CpG2	−0.048	−0.012	+0.066	+0.020	−0.018	−0.066	+0.099	−0.134^a^
CpG3	−0.032	−0.020	+0.024	−0.036	+0.031	+0.019	−0.003	−0.051
CpG4	−0.085	−0.006	+0.102	+0.024	+0.016	−0.084	−0.021	−0.161^a^
CpG5	+0.016	+0.041	−0.030	−0.013	+0.015	−0.031	−0.037	−0.011
CpG6	+0.021	+0.014	+0.050	+0.047	−0.091	−0.107	+0.037	−0.102
CpG7	−0.088	+0.096	+0.126	+0.094	−0.195^b^	−0.175^a^	+0.014	−0.138^a^
CpG average	−0.061	+0.059	+0.112	+0.061	−0.125	−0.169^a^	+0.021	−0.184^b^

All data are adjusted for age and Hamilton Depression Rating Scale score.

a p-value<0.05;

b p-value<0.01.

## Discussion

Principal findings in this longitudinal study of a post-stroke cohort were that the *BDNF* val66met polymorphism was independently associated with acute and long-term poor outcomes, and with worsening of physical disability and cognitive function. Higher *BDNF* promoter methylation status was independently associated with long-term but not with acute outcome, and was significantly associated with the worsening of physical disability and cognitive function particularly over one year. No significant effects on stroke severity measured by the NIHSS were observed. No significant genotype-methylation interactions were found.

BDNF is the most abundant neurotrophin and regulates neuronal plasticity within the brain [1]. The crucial role of BDNF in stroke recovery has been repeatedly suggested in animal studies in that brain BDNF is increased in cerebral ischemia [21], [22], motor function improvement is associated with BDNF upregulation [23], and BDNF administration improves sensory motor recovery [3], [24], [25], whereas BDNF blockade prevents recovery [5], [26]. However, this hypothesis has been controversial in human research, in that no significant increase in blood BDNF levels were found after stroke [27], and associations between the *BDNF* met allele and ischemic stroke outcomes were not found to be significant [6], [7], although significant associations have been found in aneurismal subarachnoid haemorrhage [8], [9]. To our knowledge, our study is the first to report significant associations between the met allele and outcomes over 1 year after ischemic stroke. Ethnic differences in the risk allele frequency may underlie the positive findings. Our sample had higher *BDNF* met allele (49%) compared to reports from Western populations (18–21%) [6], [8], [9], although allele frequencies were similar to reports from other Asian populations [28]. In addition, the *BDNF* met allele frequency of this stroke patients were similar to that from a population based study of a Korean elderly (47%) [29]. These may have increased the statistical power to detect associations, and also raise the question of public health relevance in East Asian populations in terms of the increased genetic vulnerability to poor outcomes after stroke.

CpG methylation status at promoter regions on the *BDNF* gene may influence stroke outcomes, since it also regulates BDNF release [10], and so we took the opportunity to test the hypothesis within a defined post-stroke cohort. To our knowledge, ours is the first report of *BDNF* methylation status with respect to stroke outcomes. As postulated, higher methylation percentages were independently associated with outcomes particularly at 1 year after stroke. These are consistent with recent findings that *BDNF* methylation is associated with memory consolidation [30] and exercise related neuronal plasticity [31] in rats, although these were not stroke models.

It is noteworthy that measures of higher *BDNF* methylation status were more strongly associated with longterm stroke outcomes, while the *BDNF met* allele was associated with both acute and longterm outcomes. *BDNF* has shown early genomic response following ischemic injury in rat brain [32], and therefore the influence of *BDNF* genotype on BDNF secretion might be initiated at the acute phase of stroke. Furthermore, given that the *BDNF met* allele is associated with reduced activity-dependent secretion of BDNF [4], functional deficiency at the chronic phase of stroke might also be associated with less BDNF secretion. However, it is not known how *BDNF* methylation status influences BDNF secretion over time after stroke, and it is important to bear in mind that there were no significant genotype-methylation interactions (Table 5) and no direct associations between *BDNF* genotype and methylation percentages (Table 6) in our sample. This is discordant with a previous study which reported the *BDNF* methylation status was dependent on genotype, and consequently had a differential effect on major psychosis (Mill et al. 2008) [11]. However, further research into the role of *BDNF* in stroke patients is indicated.

Our study has several strengths, as well as being the first to report on associations between *BDNF* methylation status and stroke outcomes. Stroke outcomes were assessed at a similar time point (two weeks and one year after stroke) in all participants, which reduced the risk of error arising from heterogeneous examination times. Participants were recruited consecutively from all eligible patients with a recent stroke at the study hospital, which reduced the likelihood of selection bias and increased the potential generalizability. In addition, a range of covariates were considered in the analyses, and the follow-up rate was reasonable and not apparently differential with respect to risk factors of interest.

Our study also has some limitations. Blood samples were obtained in only 68% of the total stroke sample in the parent study, although there were no significant differences in demographic and clinical characteristics between those with and without samples. Second, methylation status was investigated with only one CpG island of the *BDNF* gene. Further studies of other CpG islands for this gene, and for genome-wide DNA methylation are therefore needed. Third, the *BDNF* promoter methylation profile could be tissue specific. Although our results on methylation status in genomic DNA isolated from leukocytes with longterm stroke outcomes have some prognostic value, it is not clear whether this would be the relevant tissue/cell type to infer BDNF expression of most importance for stroke. Fourth, the sample size was limited for detecting associations particularly with *BDNF* genotype and gene-methylation interactions. Another important consideration is that participants with severe cognitive impairment or aphasia were excluded due to the particular study design, and therefore the present findings can only be assumed to apply to people with mild to moderate stroke severity without these deficits. Lack of associations with the scores on NIHSS might be related to this issue.

In conclusion, our findings support a role for BDNF in poststroke recovery and have several potential implications. Considering the significant morbidity poststroke, more careful evaluation and management are indicated for those with increased genetic vulnerability, particularly for East Asian populations who had higher met allele frequency. The DNA promoter methylation profile of the *BDNF* might be a prognostic biomarker for long-term stroke outcomes. However as with any first report, our findings need further replication by other study groups. Methylation tests might have clinical utility because they are non-invasive, DNA based analyses are convenient to conduct due to the amplifiable and stable nature of DNA, and are advantageous over blood BDNF levels which poorly accurately reflect brain BDNF status after stroke [27]. There are also potential treatment implications. Development of a new drug, which could increase BDNF release and regulate *BDNF* promoter methylation, may be helpful for enhancing stroke recovery [33]. In essence, epigenetics is at an embryonic stage, although is a potentially promising area of enquiry in stroke research. We believe that our study represents an important first step to elucidate the role of epigenetic mechanisms in stroke recovery, and as such is a reference point for future research.

## Supporting Information

Figure S1 Primary experimental data for *BDNF* val66met genotyping. Left panel: *BDNF* val66met amplificaion products using 3% agarose gel. Lane M-50 bp DNA ladder. Lane1-4-PCR-BDNF product of 171 bp. Right panel: *BDNF* val66met genotyping using 3% agarose gel. Lane M-50 bp DNA ladder. *BDNF* val/val genotype was presented with 99 and 72 bp (lane 2, 5, and 10); val/met genotype with 171, 99, and 72 bp (lane 1, 3, 4, 6, 8, and 9); and met/met genotype with 171 bp bands (lane 7 and 11).
(TIF)

Figure S2 Primary experimental data for *BDNF* DNA promoter methylation analysis. Seven CpG sites methylation percentages of the *BDNF* promoter region in a patient sample.
(TIF)

## References

[1] Lu B (2003) BDNF and activity-dependent synaptic modulation. Learn Mem 10: 86–98. 12663747 10.1101/lm.54603 PMC5479144

[2] Kurozumi K, Nakamura K, Tamiya T, Kawano Y, Kobune M, et al (2004) BDNF gene-modified mesenchymal stem cells promote functional recovery and reduce infarct size in the rat middle cerebral artery occlusion model. Mol Ther 9: 189–197. 14759803 10.1016/j.ymthe.2003.10.012

[3] Schabitz WR, Steigleder T, Cooper-Kuhn CM, Schwab S, Sommer C, et al (2007) Intravenous brain-derived neurotrophic factor enhances poststroke sensorimotor recovery and stimulates neurogenesis. Stroke 38: 2165–2172. 17510456 10.1161/STROKEAHA.106.477331

[4] Egan MF, Kojima M, Callicott JH, Goldberg TE, Kolachana BS, et al (2003) The BDNF val66met polymorphism affects activity-dependent secretion of BDNF and human memory and hippocampal function. Cell 112: 257–269. 12553913 10.1016/s0092-8674(03)00035-7

[5] Qin L, Kim E, Ratan R, Lee FS, Cho S (2011) Genetic Variant of BDNF (Val66Met) Polymorphism Attenuates Stroke-Induced Angiogenic Responses by Enhancing Anti-Angiogenic Mediator CD36 Expression. J Neurosci 31: 775–783. 21228186 10.1523/JNEUROSCI.4547-10.2011 PMC3308129

[6] Cramer SC, Procaccio V (2012) GAIN Americas GAIN International Study Investigators (2012) Correlation between genetic polymorphisms and stroke recovery: Analysis of the GAIN Americas and GAIN International Studies. Eur J Neurol 19: 718–724. 22221491 10.1111/j.1468-1331.2011.03615.x

[7] Manso H, Krug T, Sobral J, Albergaria I, Gaspar G, et al (2012) Evidence for epistatic gene interactions between growth factor genes in stroke outcome. [published online ahead of print January 10, 2010] Eur J Neurol doi:10.1111/j.1468-1331.2011.03625.x. 10.1111/j.1468-1331.2011.03625.x 22233184

[8] Siironen J, Juvela S, Kanarek K, Vilkki J, Hernesniemi J, et al (2007) The Met allele of the BDNF Val66Met polymorphism predicts poor outcome among survivors of aneurysmal subarachnoid hemorrhage. Stroke 38: 2858–2860. 17761923 10.1161/STROKEAHA.107.485441

[9] Vilkki J, Lappalainen J, Juvela S, Kanarek K, Hernesniemi JA, et al (2008) Relationship of the Met allele of the brain-derived neurotrophic factor Val66Met polymorphism to memory after aneurysmal subarachnoid hemorrhage. Neurosurgery 63: 198–203. 18797348 10.1227/01.NEU.0000320382.21577.8E

[10] Martinowich K, Hattori D, Wu H, Fouse S, He F, et al (2003) DNA methylation related chromatin remodeling in acticity-dependent BDNF gene regulation. Science 302: 890–893. 14593184 10.1126/science.1090842

[11] Mill J, Tang T, Kaminsky Z, Khare T, Yazdanpanah S, et al (2008) Epigenomic profiling reveals DNA-methylation changes associated with major psychosis. Am J Hum Genet 82: 696–711. 18319075 10.1016/j.ajhg.2008.01.008 PMC2427301

[12] Kim JM, Stewart R, Bae KY, Kim SW, Kang HJ, et al (2012) Serotonergic and BDNF genes and risk of depression after stroke. J Affect Disord 136: 833–840. 22014446 10.1016/j.jad.2011.09.029

[13] Sacco RL, Adams R, Albers G, Alberts MJ, Benavente O, et al (2006) Guidelines for prevention of stroke in patients with ischemic stroke or transient ischemic attack: a statement for healthcare professionals from the American Heart Association/American Stroke Association Council on Stroke: co-sponsored by the Council on Cardiovascular Radiology and Intervention: the American Academy of Neurology affirms the value of this guideline. Stroke 37: 577–617. 16432246 10.1161/01.STR.0000199147.30016.74

[14] Folstein MF, Fostein SE, McHugh PR (1975) “Mini-Mental State”: a practical method for grading the cognitive state of patients for the clinician. J Psychiatr Res 12: 189–198. 1202204 10.1016/0022-3956(75)90026-6

[15] van Swieten JC, Koudstaal PJ, Visser MC, Schouten HJ, van GJ (1988) Interobserver agreement for the assessment of handicap in stroke patients. Stroke 19: 604–607. 3363593 10.1161/01.str.19.5.604

[16] Kasner SE, Chalela JA, Luciano JM, Cucchiara BL, Raps EC, et al (1999) Reliability and validity of estimating the NIH stroke scale score from Medical records. Stroke 30: 1534–1537. 10436096 10.1161/01.str.30.8.1534

[17] Mahoney FI, Barthel DW (1965) Functional evaluation: the Barthel Index. MD State Med J 14: 61–65. 14258950

[18] Devlin AM, Brain U, Austin J, Oberlander TF (2010) Prenatal exposure to maternal depressed mood and the MTHFR C677T variant affect SLC6A4 methylation in infants at birth. PLoS ONE 5: e12201. 20808944 10.1371/journal.pone.0012201 PMC2922376

[19] Roth TL, Lubin FD, Funk AJ, Sweatt JD (2009) Lasting epigenetic influence of early-life adversity on the BDNF gene. Biol Psychiatry 65: 760–769. 19150054 10.1016/j.biopsych.2008.11.028 PMC3056389

[20] Hamilton M (1960) A rating scale for depression. J Neurol Neurosurg Psychiatry 23: 56–62. 14399272 10.1136/jnnp.23.1.56 PMC495331

[21] Bejot Y, Mossiat C, Giroud M, Prigent-Tessier A, Marie C (2011) Circulating and brain BDNF levels in stroke rats. Relevance to clinical studies. PLoS One 6: e29405. 22195050 10.1371/journal.pone.0029405 PMC3241711

[22] Berger C, Schabitz WR, Wolf M, Mueller H, Sommer C, et al (2004) Hypothermia and brain-derived neurotrophic factor reduce glutamate synergistically in acute stroke. Exp Neurol 185: 305–312. 14736512 10.1016/j.expneurol.2003.10.008

[23] Murphy TH, Corbett D (2009) Plasticity during stroke recovery: from synapse to behaviour. Nat Rev Neurosci 10: 861–872. 19888284 10.1038/nrn2735

[24] Schabitz WR, Berger C, Kollmar R, Seitz M, Tanay E, et al (2004) Effect of brain derived neurotrophic factor treatment and forced arm use on functional motor recovery after small cortical ischemia. Stroke 35: 992–997. 14988579 10.1161/01.STR.0000119754.85848.0D

[25] Muller HD, Hanumanthiah KM, Diederich K, Schwab S, Schabitz WR, et al (2008) Brain-derived neurotrophic factor but not forced arm use improves long-term outcome after photothrombotic stroke and transiently upregulates binding densities of excitatory glutamate receptors in the rat brain. Stroke 39: 1012–1021. 18239176 10.1161/STROKEAHA.107.495069

[26] Ploughman M, Windle V, MacLellan CL, White N, Dore JJ, et al (2009) Brain-derived neurotrophic factor contributes to recovery of skilled reaching after focal ischemia in rats. Stroke 40: 1490–1495. 19164786 10.1161/STROKEAHA.108.531806

[27] Di Lazzaro V, Profice P, Pilato F, Dileone M, Florio L, et al (2007) BDNF plasma levels in acute stroke. Neurosci Lett 422: 128–130. 17590513 10.1016/j.neulet.2007.06.001

[28] Kunugi H, Iijima Y, Tatsumi M, Yoshida M, Hashimoto R, et al (2004) No association between the Val66Met polymorphism of the brain-derived neurotrophic factor gene and bipolar disorder in a Japanese population: a multicenter study. Biol Psychiatry 56: 376–378. 15336520 10.1016/j.biopsych.2004.06.017

[29] Kim JM, Stewart R, Kim SW, Yang SJ, Shin IS, et al (2007) Interactions between life stressors and susceptibility genes (5-HTTLPR and BDNF) on depression in Korean elders. Biol Psychiatry 62: 423–428. 17482146 10.1016/j.biopsych.2006.11.020

[30] Lubin FD, Roth TL, Sweatt JD (2008) Epigenetic regulation of BDNF gene transcription in the consolidation of fear memory. J Neurosci 28: 10576 10586. 18923034 10.1523/JNEUROSCI.1786-08.2008 PMC3312036

[31] Gomez-Pinilla F, Zhuang Y, Feng J, Ying Z, Fan G (2011) Exercise impacts brain-derived neurotrophic factor plasticity by engaging mechanisms of epigenetic regulation. Eur J Neurosci 33: 383–390. 21198979 10.1111/j.1460-9568.2010.07508.x PMC3256007

[32] Rickhag M, Teilum M, Wieloch T (2007) Rapid and long-term induction of effector immediate early genes (BDNF, Neuritin and Arc) in peri-infarct cortex and dentate gyrus after ischemic injury in rat brain. Brain Res 1151: 203–210. 17397810 10.1016/j.brainres.2007.03.005

[33] Nagahara AH, Tuszynski MH (2011) Potential therapeutic uses of BDNF in neurological and psychiatric disorders. Nat Rev Drug Discov 10: 209–219. 21358740 10.1038/nrd3366

