# Supplementary figures and images for: Associations of BDNF Genotype and Promoter Methylation with Acute and Long-Term Stroke Outcomes in an East Asian Cohort
Source: PLoS One. 2012 Dec 11;7(12):e51280. doi: 10.1371/journal.pone.0051280 (PMC3519835; doi:10.1371/journal.pone.0051280)

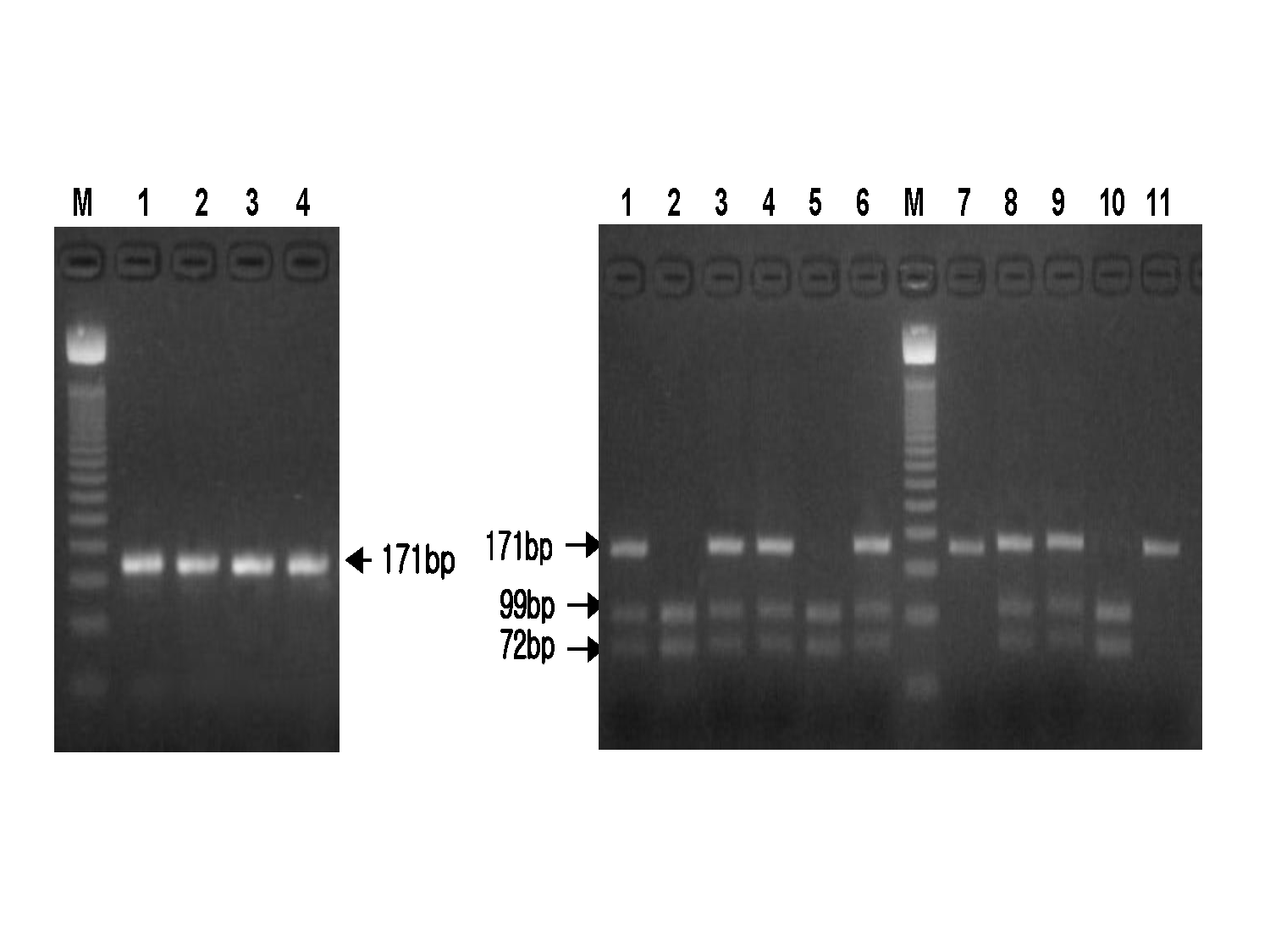

Supplement: Figure S1 — Primary experimental data for BDNF val66met genotyping. Left panel: BDNF val66met amplificaion products using 3% agarose gel. Lane M-50 bp DNA ladder. Lane1-4-PCR-BDNF product of 171 bp. Right panel: BDNF val66met genotyping using 3% agarose gel. Lane M-50 bp DNA ladder. BDNF val/val genotype was presented with 99 and 72 bp (lane 2, 5, and 10); val/met genotype with 171, 99, and 72 bp (lane 1, 3, 4, 6, 8, and 9); and met/met genotype with 171 bp bands (lane 7 and 11). (TIF) [file pone.0051280.s001.tif]

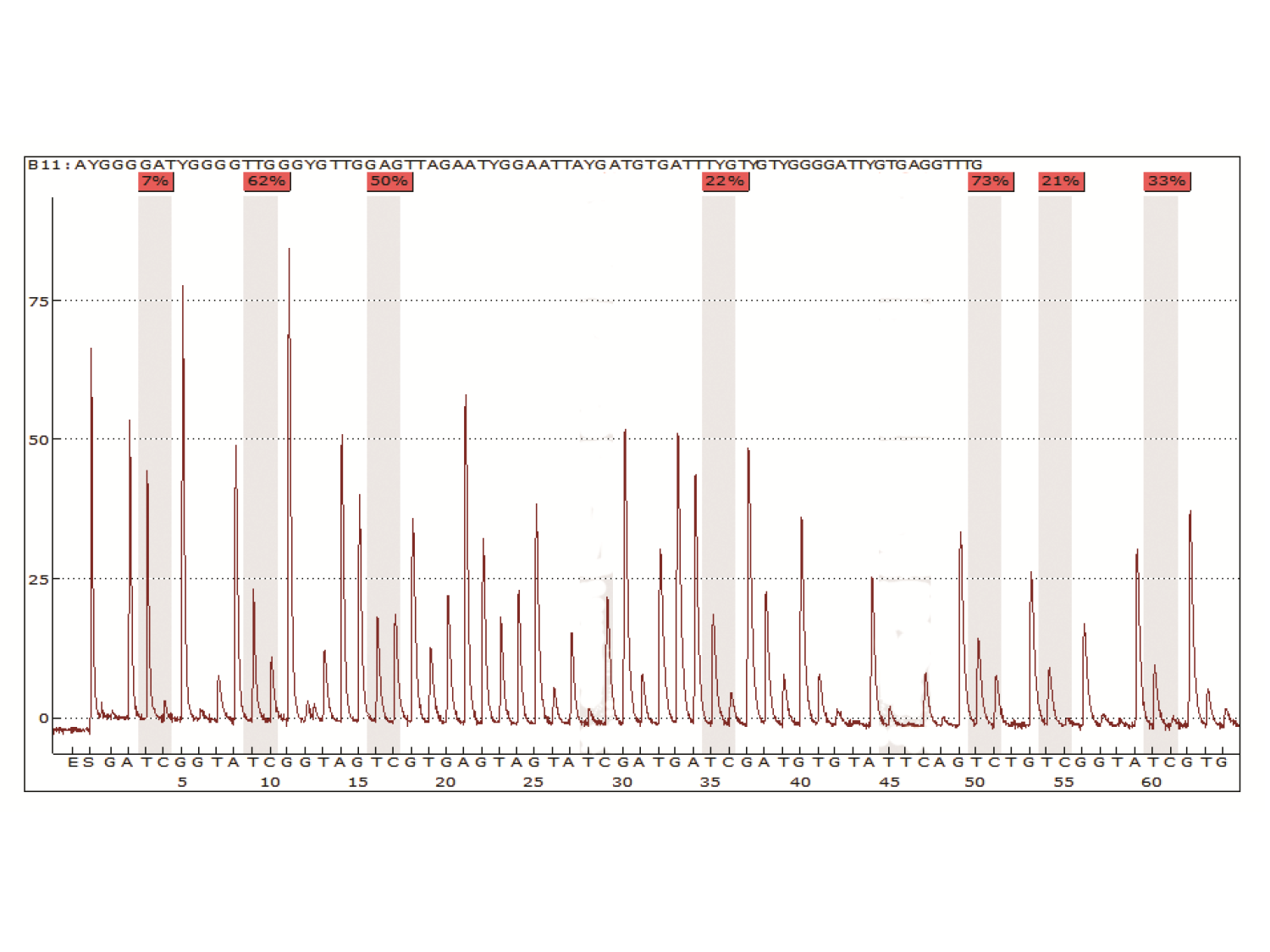

Supplement: Figure S2 — Primary experimental data for BDNF DNA promoter methylation analysis. Seven CpG sites methylation percentages of the BDNF promoter region in a patient sample. (TIF) [file pone.0051280.s002.tif]
